# Effect of mouthwashes on the composition and metabolic activity of oral biofilms grown in vitro

**DOI:** 10.1007/s00784-016-1876-2

**Published:** 2016-06-23

**Authors:** Mercedes Fernandez y Mostajo, Rob A. M. Exterkate, Mark J. Buijs, Wim Crielaard, Egija Zaura

**Affiliations:** Department of Preventive Dentistry, Academic Centre for Dentistry Amsterdam (ACTA), University of Amsterdam and Free University Amsterdam, Gustav Mahlerlaan 3004, 1081 LA Amsterdam, The Netherlands

**Keywords:** Biofilm, Microcosm, Organic acids, Mouthwashes, Antimicrobials

## Abstract

**Objective:**

The aim of this study was to determine the effect of an oxygenating mouthwash compared to two other established mouthwash products on bacterial composition and metabolic activity of oral biofilms in vitro.

**Material and methods:**

Twelve healthy subjects participated as donors. Plaque-saliva mixture inoculated biofilms were grown and treated with 3 different chemotherapeutic mouthwashes [amine fluoride/stannous fluoride (MD), oxygenating agent (AX), chlorhexidine 0.12 % (PA), and water (W)]. Effects of treatments were assessed on biofilm composition (16S rRNA gene amplicon sequencing), production of organic acids (formate, acetate, lactate, propionate, butyrate using capillary electrophoresis), and viability of the remaining biofilm (CFUs).

**Results:**

Microbial profiles of biofilms clustered per inoculum donor and were dominated by the genera *Veillonella*, *Streptococcus*, and *Prevotella*. Microbial diversity was only reduced after PA treatment. Significant changes in composition occurred after treatment with AX, resulting in lower proportions of *Veillonella* and higher proportions of non-mutans streptococci. Production of all organic acids after PA and lactate after MD was significantly lower as compared to W. AX resulted in reduction of acetate, butyrate, and propionate and increase in lactate production (*p* < 0.05). Viable counts were significantly lower after PA and AX treatments compared to W, while no significant reduction was observed after MD.

**Conclusions:**

All studied mouthwashes affected the in vitro biofilms differently. The effects of the AX treatment were the most prominent which resulted in changes of the bacterial composition and metabolism.

**Clinical implications:**

Awareness by the dental team that mouthwashes can change the bacterial composition and metabolism is important when advising its use.

## Introduction

Dental plaque biofilms are communities of microbial cells in the oral cavity that are embedded in an extracellular polymeric matrix. Oral biofilms are among the most complex microbial communities in nature. Dental plaque affects all humans worldwide. Failure to deal with plaque in the early stage of formation may lead to the development of oral diseases of which the most prevalent are dental caries, gingivitis, and periodontitis [1]. Since the mouth is the gateway to the human body, establishing and maintaining oral health is important.

Various strategies have been employed to reduce or eliminate dental biofilms. Mechanical disruption of dental plaque biofilms such as toothbrushing is worldwide the most common approach. The effectiveness of this is however limited [2, 3]. Therefore, also chemotherapeutic strategies to disrupt these biofilms have been considered, such as the use of mouthwashes. Over the past decades, the use of these has become customary, usually following mechanical plaque biofilm control [4].

Currently, there is a wide range of over the counter mouthwashes products available, containing various active ingredients with for each of these specific indications. It is the role of the dental teams to advice the use of mouthwashes when indicated, which is justified when its efficacy has been proven by studies based on clinical evidence.

A recent meta-review [4] has shown that, to date, the scientific body of evidence for the use of specific agents such as chlorhexidine is substantial. However, for most commercially available mouthwashes, the scientific evidence is considered to be moderate, weak, or underreported. For instance, the underlying data retrieved in a systematic review for oxygenating agents (OAs) did not allow for a meta-analysis [5].

Serrano and colleagues [6] have analyzed the scientific evidence for the efficacy of adjunctive anti-plaque chemical agents in managing gingivitis. These authors concluded that the use of specific agents showed statistically significant improvements in terms of gingival, bleeding and plaque indices. OAs were not included in their meta-analysis.

Nevertheless, most mouthwashes, among which also OAs, are available without a prescription and their use is a common practice. Oxygenating agents, such as hydrogen peroxide (H_2_O_2_), buffered sodium peroxyborate, and peroxycarbonate, have been recommended for short-term use as disinfectants [4, 5]. Recently a mouthwash containing an oxygenating agent (peroxoborate) has been introduced in the market and the result of a pilot study has shown that this product has the potential for selective inhibition of oral bacteria. Twice-daily exposure for 1 week to this mouthwash resulted in a shift in the microbial composition towards a less diverse and less mature plaque [7].

Oral microbes contribute to the healthy homeostasis within the oral cavity [8]. Therefore, besides addressing a clinical efficacy of a mouthwash, it is important to investigate its effects on the oral microbiome [9]. For this purpose, in vitro biofilm models offer an approach to elucidate the microbiological and ecological effect of potential products at an early stage of product development [10–15].

The aims of this study were to assess and determine the effect of an oxygenating mouthwash compared to two other established and commercially available mouthwashes products and a negative water control on the bacterial composition and metabolic activity of oral biofilms in vitro.

The null hypothesis was that the treatment effect of an oxygenating and a fluoride-based mouthwash will not differ in terms of bacterial composition and metabolic activity than compared to a negative control (water) or a positive control (chlorhexidine).

## Material and methods

### Selection of inoculum donors

Twelve individuals participated as donors of saliva and dental plaque after reading and signing an informed consent form. All donors were in good general health and had not been exposed to antibiotics and professional dental prophylaxis within the previous 3 months. Exclusion criteria were visible caries or a Dutch periodontal screening index (DPSI) of smaller or equal to score 3 minus (which translates to periodontal pockets ≤5 mm in absence of gingival recession) [16]. Prior to sample collection, subjects were asked not to perform any oral hygiene 24 h before the appointment and to refrain from any food or drink consumption for at least 2 h before sampling [17].

Ethical approval of the protocol related to plaque-saliva collection and experimental research was provided by the Medical Ethical Committee from the Vrije Universiteit Medical Center Amsterdam (reference 2011/236).

### Sampling

Sampling took place in the morning between 08:30 and 09:30. Supragingival dental plaque was collected with sterile plastic curettes along the gingival margin of all buccal and lingual surfaces in the second and fourth quadrants. The samples were placed in a previously weighed Eppendorf vial containing 100 μl of cysteine peptone water (CPW), vortexed for 30 s, and briefly centrifuged. Then, the vials were weighed again and the difference in weight was calculated. CPW was added normalizing to a proportion of 10 mg plaque: 1.3 ml CPW and stored on ice. Subsequently, participants chewed on a piece of a parafilm for 1 min. Approximately 1 ml of -stimulated saliva was collected in a sterile universal vial being kept on ice.

### Biofilm model and production of the biofilms

An active attachment biofilm model was used as previously described [11]. In brief, 24-well polystyrene culture plates (Greiner Bio-One, Alphen a/d. Rijn, the Netherlands) were used. The lid of the plate was replaced by a custom-made stainless-steel lid, onto which 24 nylon clamps were fixed allowing for various substrata to be inserted. The clamps were positioned in such a way that the inserted substrata would fit into the wells of the culture plate. Since the substrata were positioned vertically, bacterial attachment plays a key role in this model. By transferring the lid with the substrata to a new plate, the medium could be refreshed. In this study, standardized 12-mm hydroxyapatite (HA) disks (Himed, Old Bethpage, USA) were used as substratum. The assembled model was autoclaved at 121 °C.

McBain medium [18] with 50 mmol/l PIPES at pH 7 [19] was used. After autoclaving, the medium was supplemented with filter-sterilized 0.2 % sucrose and sterile heat-inactivated 5 % fetal bovine serum (FBS, F4135, Sigma-Aldrich, USA).

The suspension of dental plaque samples was dispersed using a sonicator (Vibra Cell, Sonics & Materials Inc., Newtown, CT) for 3 s with 1-s pulses at amplitude of 40 W and vortex-mixing for 30 s just before inoculation. The inoculation medium was immediately prepared using a mixture of dental plaque 1:15 (1.25 ml of suspension in 20 ml of CPW) and saliva 1:50 (0.4 ml of saliva in 20 ml of CPW) as inoculum. Next 1.5 ml of the inoculation medium was added to each well of a standard polystyrene 24-well plate (multiwell plates; Greiner Bio One, Alphen aan den Rijn, The Netherlands). For each donor, an individual plate was used. The model was subsequently incubated anaerobically (10 % CO_2_, 10 % H_2_, and 80 % N_2_) at 37 °C. After an initial 24 h of incubation, the medium was refreshed with sterile medium twice a day based on a schedule of 8 and 16 h, up to 96 h.

To analyze the bacterial composition of the inocula, 100 μl of the plaque-saliva suspension was stored at −20 °C for DNA extraction and analysis.

### Treatment of the biofilms

After 96 h, the biofilms were treated by transferring them to a new plate containing the treatment solution (1.8 ml/well) for 10 min at room temperature with one of the following compounds:

1. Perioaid® (Dentaid Benelux, Houten, the Netherlands) containing 0.12 % chlorhexidine (PA).
2. Meridol® (GABA International, Basel, Switzerland), an amine fluoride/stannous fluoride (AmF/SnF_2_)-containing mouthwash (MD).
3. Ardox-X-technology®, commercially known as O7-active (NGen Oral Pharma N.V., Curacao) an oxygenating agent (AX).
4. Sterile demineralized water as a negative control.

Triplicate biofilms were treated with the abovementioned compounds and a negative water control resulting in four treatment groups for each donor.

Following treatment, biofilms were rinsed with CPW (1.9 ml/well) moving the lid 10 times up and down in the plate [19]. This rinsing procedure was repeated 3 times with fresh CPW to remove excess treatment solution.

### Biofilm incubation for acid formation

Subsequently, the lid was placed on a plate containing 1.5 ml buffered peptone water (BPW) with 0.2 % sucrose per well and incubated anaerobically for 3 h at 37 °C. After the incubation, BPW was collected and stored at −80 ° C.

### Harvesting of the biofilms

Each HA disk with biofilm was removed using sterile forceps and placed in a sterile vial containing 2 ml of phosphate-buffered saline (PBS). All samples were kept on ice, dispersed by sonication for 2 min at 1 s pulsations at the amplitude of 40 W (Vibra Cell; Sonics & Materials Inc., Newtown, CT) and vortexed for 30 s.

### Determination of number of CFUs

Serial dilutions of the dispersed biofilms in PBS were made and plated on trypticase soy agar blood (TSAB) plates for total viable counts. The plates were incubated for 7 days at 37 °C under anaerobic conditions (10 % CO_2_, 10 % H_2_, and 80 % N_2_), and the number of colony-forming unit (CFU) counts was determined.

### Propidium monoazide (PMA) treatment

To assure that only DNA from the cells with intact membranes (undamaged cells) was processed for sequencing, the dispersed biofilms were treated with PMA as described previously [19]. In brief, 2.5 μl PMA (Biotum Inc., Hayward, Calif., USA) was added to 500 μl suspended biofilm, incubated in the dark for 5 min, and then exposed to intense light for 2 min using a 650 W halogen lamp placed 25 cm from the samples. The samples were kept on ice during this procedure.

### Organic acid analysis

The concentration of organic acids in BPW following incubation with sucrose was determined by capillary electrophoresis (Waters Capillary Ion Analyzer Milford, Mass, USA) as described previously [20]. As an internal standard, 0.12 mmol/l oxalate was included in all samples. Succinate, formate, acetate, lactate, propionate, and butyrate were determined and expressed as mmol acid/disk. The total concentration of acid excreted by the biofilms to the environment (BPW) was calculated as the sum of all acids per biofilm.

### DNA extraction, amplicon preparation, and sequencing data analysis

DNA from the inocula and biofilms was extracted as previously described [21]. Barcoded amplicon libraries of the small subunit ribosomal RNA gene hypervariable region V5–V7 were generated for each of the individual samples, pooled and sequenced using Genome Sequencer FLX Titanium system (Roche, Basel, Switzerland) as described previously [22]. The sequencing data were processed using Quantitative Insights Into Microbial Ecology (QIIME) [23] version 1.5.0 as described previously [22].

To allow comparisons among different samples, the dataset containing Operational Taxonomic Units (OTUs) was randomly subsampled at an equal depth. Hierarchical clustering based on the Bray-Curtis similarity index (BC) was used to assess the similarities among the biofilms per group—a non-linear coefficient between every pair of OTUs. Shannon diversity index was calculated, which takes into account the abundance of each OTU, as well as the number of OTUs. To visualize microbial profile data, non-metric multidimensional plots (n-MDS) based on the BC were used.

In addition, taxonomy of the representative sequences of the most abundant OTUs was identified by microbial nucleotide BLAST search against a Human Oral Microbiome Database (HOMD) reference set (version 13.2). The possible alternatives for the species identification were then provided.

### Statistical analysis

Statistical analyses were performed on median values of triplicate biofilm data. Non-metric multidimensional scaling (nMDS) plots were based on the Bray-Curtis coefficient to visualize similarity between samples. Stress <0.2 (Kruskal’s stress formula 1) was used as an acceptable threshold [24]. Similarity percentage (SIMPER) was used to identify the OTUs with the highest contribution to dissimilarity between the treatments. The differences in composition between treatment groups were assessed using one-way permutational multivariate analysis of variance (PERMANOVA). The *p* values were corrected for multiple comparisons using Bonferroni correction. *p* values <0.05 were considered statistically significant. All calculations were performed using PAST version 3.0 [25].

The software package IBM SPSS Statistics version 20.0 (2011, IBM Corp., Armonk, NY, USA) was used to perform all other statistical analyses. For comparisons among the treatment groups based on CFU counts, the median of triplicate biofilms was calculated, based on which the percentage of the remaining biofilm relative to the water-treatment was determined. Almost all groups were normally distributed (Shapiro-Wilk test) except for the AX group. Pairwise comparisons were performed using Paired sample *t* tests, and corrected for multiple comparisons using Bonferroni corrections.

Differences among the organic acid profiles per treatment group were analyzed regardless of the donor using one-way-ANOVA with Games-Howel post-hoc test; *p* value corrected for multiple comparisons. Additionally, in order to determine relationships between the concentration of organic acids and the relative abundance of the most abundant genera (Table 1), a Pearson correlation was calculated.

**Table 1:** The relative proportion at genus level per mouthwash compared to the water treatment (*n* = 12)

Genus (OTU ID)		Mean %	SD	*p value*
*Veillonella* ^a^ (OTU 190)	Water	62.2	(10)	
	MD	64.2	(10)	*0.081*
	PA	68.0	(10)	*0.034*
	AX	48.2	(20)	*0.030*
*Streptococcus* ^a^ (OTU 20)	Water	15.6	(10)	
	MD	11.4	(5)	***0.001*** ^**c**^
	PA	11.1	(4)	***0.002***
	AX	28.7	(10)	*0.004*
*Prevotella* ^b^ (*OTU 296*)	Water	14	(10)	
	MD	10.9	(4)	*0.084*
	PA	7.8	(4)	*0.008*
	AX	8.7	(4)	*0.010*
*Parvimonas* ^b^ (OTU 133)	Water	2.1	(2)	
	MD	2.6	(2)	*0.099*
	PA	3.2	(3)	*0.075*
	AX	4.2	(4)	*0.015*
*Solobacterium* ^b^ (OTU 6)	Water	1.9	(2)	
	MD	1.3	(1)	*0.050*
	PA	0.9	(1)	***0.002***
	AX	0.8	(0)	*0.010*
*Megasphaera* ^b^ (OTU 88)	Water	1.5	(1)	
	MD	1.3	(1)	*0.023*
	PA	0.7	(1)	***0.002***
	AX	0.4	(0)	***0.002***

^a^Paired *t* test

^b^Wilcoxon rank test (*p* values not corrected for multiple comparisons)

^c^ *p* values (highlighted in bold) that remain significant after Bonferroni correction for multiple tests

For the comparisons of mouthwashes in relation to the bacterial composition, only the most abundant genera (on average, contributing to 94 % of reads in the water-treated biofilms) were selected. Based on their distribution (Shapiro-Wilk test), the paired *t* test or the Wilcoxon Rank test was used.

## Results

Of the 12 participants that donated dental plaque and saliva, 7 were female and 5 were male with an age range of 22–45 years. One participant was a smoker of <10 cigarettes/day (donor 4).

**Fig. 1:**
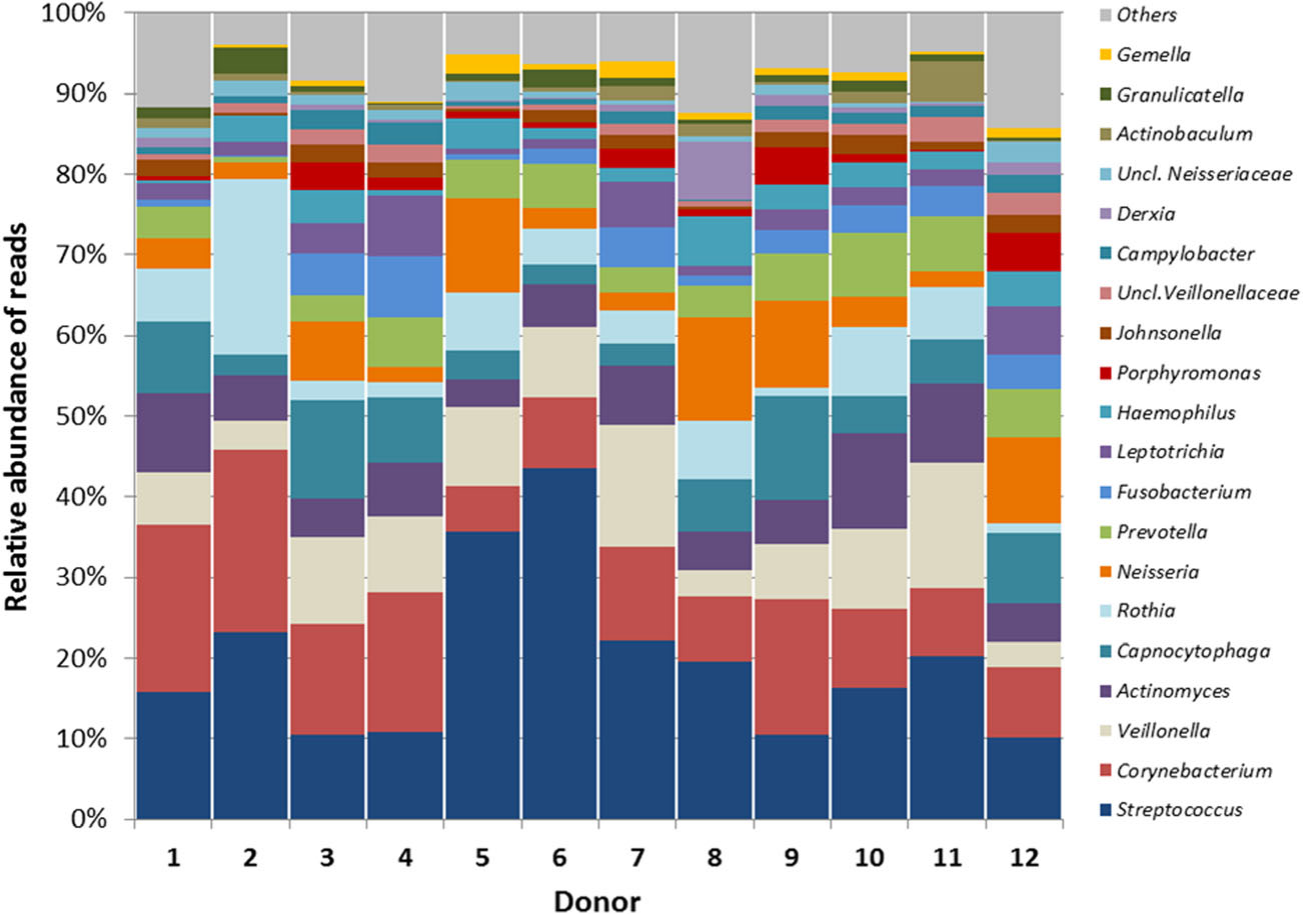
Relative abundance of the major bacterial genera in the inoculum (plaque-saliva mixture) per donor. Others—the sum of the remaining taxa

The relative abundance of bacterial genera in the inocula differed to a large extent by donor (Fig. 1). The mean Shannon diversity index of the inocula (3.05; SD 0.3) was significantly higher (*p* < 0.01) than the mean diversity index of the 96 h water-treated (W, negative control) biofilms (1.57; SD 0.5)*.*

All biofilms were dominated by genus *Veillonella* (OTU 190), *Streptococcus* (OTU 20), and *Prevotella* (OTU 296). These OTUs were blasted against a HOMD database for species-level taxonomical classification. *Veillonella* (OTU 190) was identified as *Veillonella dispar* (HOT 160)/*Veillonella rogosa* (HOT 158) at 99.7 % blast ID and as *Veillonella atypica* (HOT 524) / *Veillonella parvula* (HOT 161) at 99.7 % blast ID. *V. parvula* is phylogenetically similar to *V. dispar*; however, *V. parvula* is catalase negative whereas *V. dispar* is catalase positive.

*Streptococcus* (OTU20) was identified as *Streptococcus oralis* (HOT 707) 100 % blast ID *and as Streptococcus* species (HOT 071; HOT 070; HOT 064 and HOT 058) 100 % blast ID. OTU 296 was identified as *Prevotella melaninogenica* (HOT 469) 100 % blast ID.

The hierarchical classical cluster analysis based on the Bray-Curtis similarity index among the biofilm triplicates showed that replicate biofilms clustered together (data not shown).

**Fig. 2:**
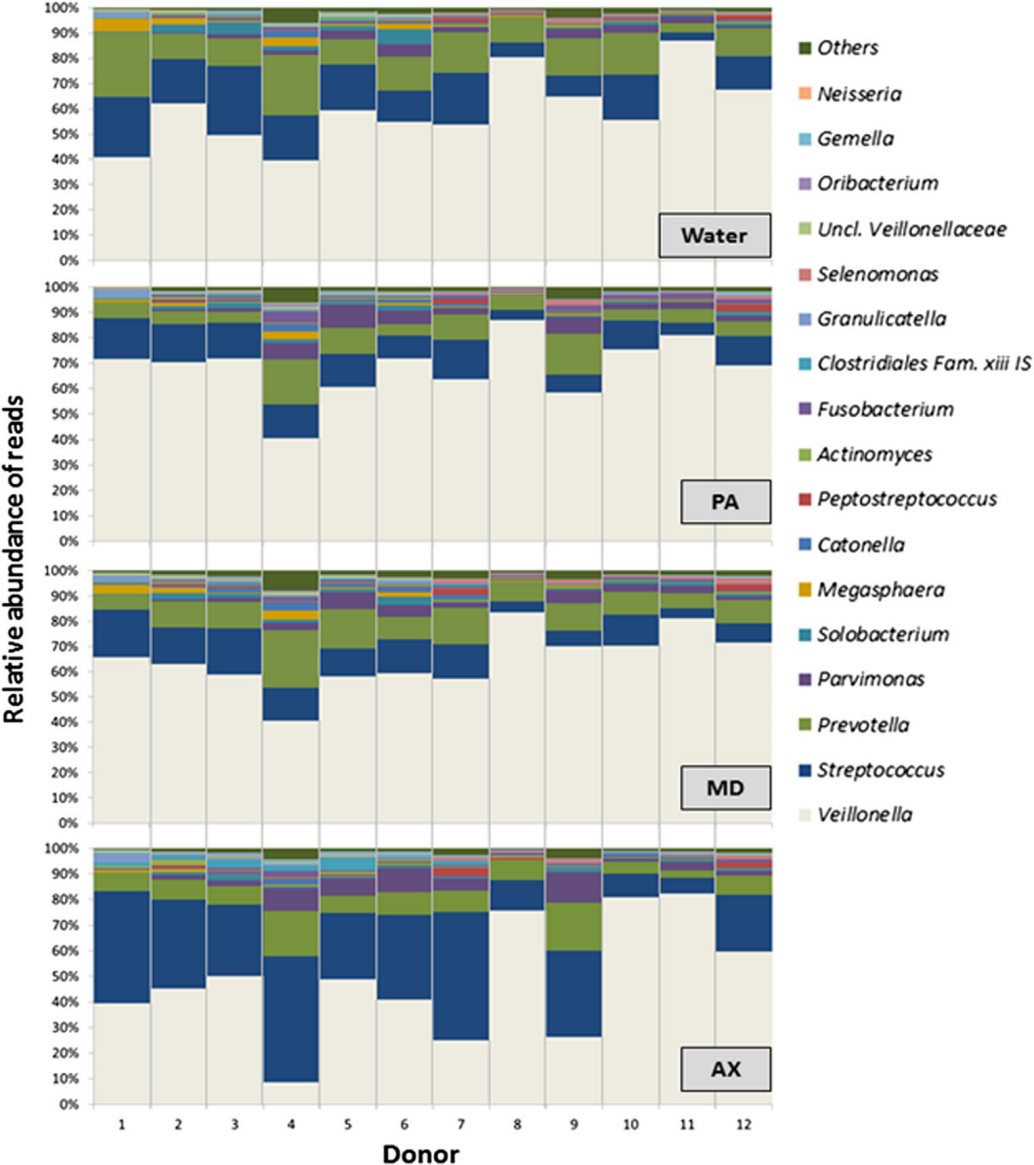
Relative abundance of major bacterial genera per donor after 10-min treatment with water, PerioAid (PA), Meridol (MD) or Ardox-X (AX). The data shown are median values from triplicate biofilms

The water-treated biofilms showed variability among donors in relative abundance of the genera *Veillonella*, *Streptococcus*, *Megasphaera*, *Peptostreptococcus*, and *Prevotella* (Fig. 2)*. Veillonella* was the most abundant genus (40–87 % of reads), followed by *Streptococcus* (3–27 %), and *Prevotella* (3–27 %) with 4–20 % of the reads belonging to other genera.

Ten-minute treatment with different compounds resulted in compositional changes in the remaining biofilm. As compared to the W-treated biofilms, the PA treatment resulted in a significantly lower diversity index (1.32, SD 0.4) compared to W-treated biofilms 1.57 (SD 0.5)*.* The relative abundance of the genus *Streptococcus*, *Solobacterium*, and *Megasphaera* was significantly lower in the PA-treated biofilms compared to the water control (Table 1, Fig. 2).

The diversity index for the MD treated biofilms (1.52, SD 0.4) was not statistically different from the water control. The relative abundance of genus *Streptococcus* was statistically lower in the MD-treated biofilms than in the water control (Table 1, Fig. 2).

Also, AX treatment had no significant effect on diversity index (1.62, SD 0.4) in the biofilms. However, genus *Megasphaera* was significantly lower in the AX-treated biofilms compared to the water control (Table 1, Fig. 2).

**Fig. 3:**
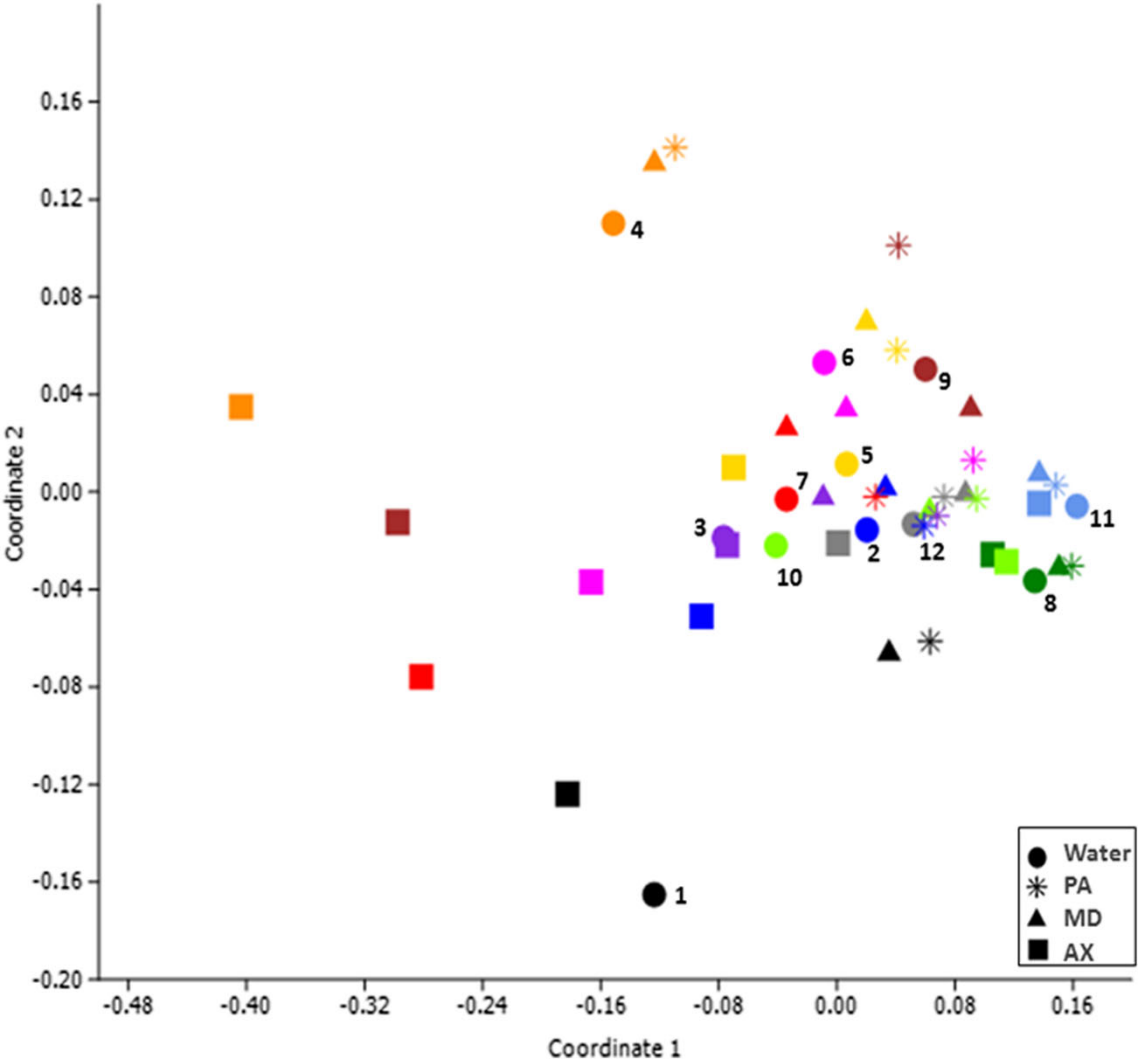
Non-metric multidimensional scaling (n-MDS) based on three-dimensional Bray-Curtis similarity (*p* = 0.001, stress = 0.07, F = 3.76), *n* = 12. *Colors* indicate individual donors. The donors’ number is shown next to the water treatment (*dot*). *Shapes* represent a treatment. Comparisons between groups were done using one-way PERMANOVA; W vs AX *p* = 0.186; W vs PA *p* = 0.394; W vs MD *p* = 1; PA vs MD *p* = 1; PA vs AX *p* = 0.015; MD vs AX *p* = 0.043

In order to assess the dissimilarities between the treatment groups, we used the non-metric multidimensional scaling (n-MDS). This analysis showed that the dissimilarities among the treatment compounds were statistically significant only when AX-treated biofilms were compared to PA or MD treated biofilms (*p* ≤ 0.05) (Fig. 3).

The effect of each of the tested compounds on the composition of the remaining biofilms had a clear pattern (Fig. 2). Variability among the biofilms originating from different donors was visually observed on a 3D plot. In particular, the respective samples from donors 8 and 11 clustered close together regardless the treatment group (Fig. 3). In contrast, biofilms from donors 2, 4, 6, 7, and 9 revealed a clear effect of the AX treatment (squares at the left side of the plot) except from donor 10 (square at the right side of the plot). The W-treated biofilms and AX-treated biofilms clustered close together in donors 1, 3, and 5.

### Treatment effect on viable counts

**Fig. 4:**
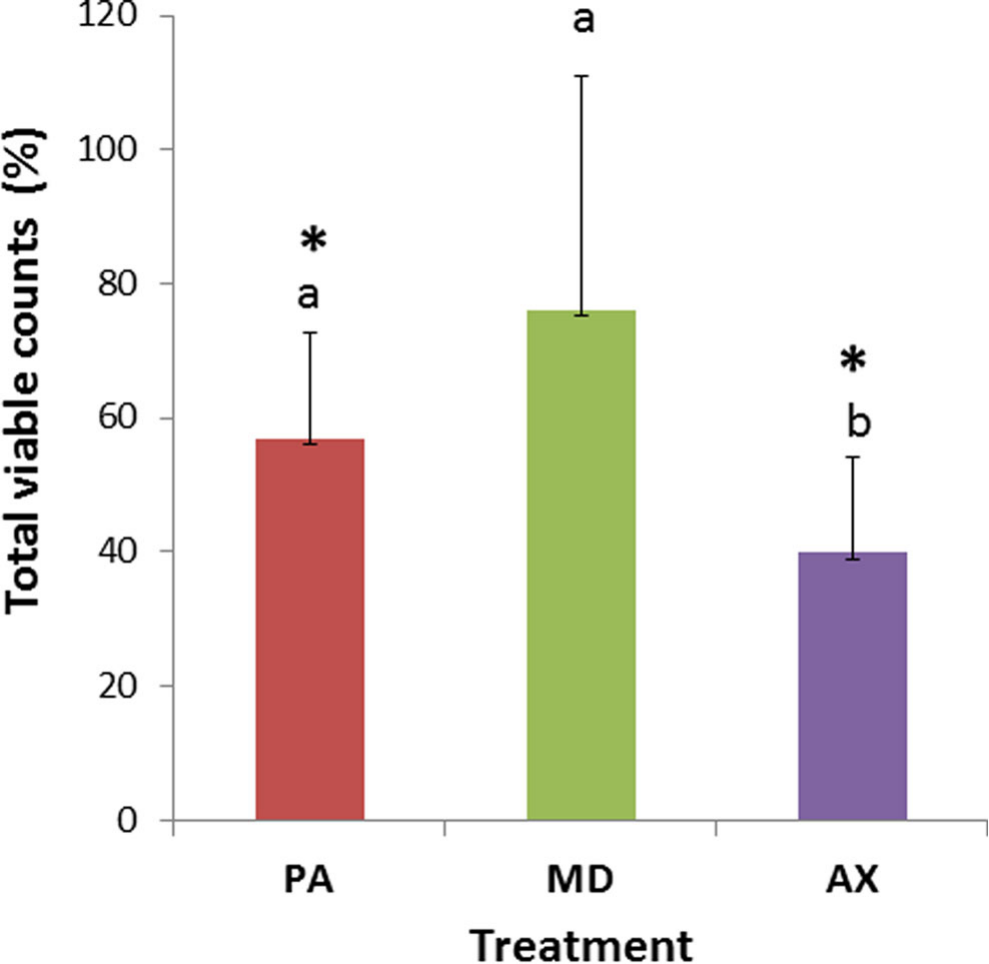
Effect of single treatment on viable counts expressed as a percentage of the water-treated control is described as the remaining biofilm. The data shown are the average and standard deviation (*n* = 12). Different *letters* indicate statistically significant differences between the treatments PerioAid (PA), Meridol (MD) or Ardox-X (AX). *statistically significant lower amount of remaining biofilms compared to the water treated control (*p* ≤ 0.05), paired sample *t* test, corrected for multiple comparisons

W-treated biofilms yielded comparable average total viable counts (5.9x10^9^ CFUs/biofilm, SD 9x10^9^) among all donors (*p* = 0.176). Compared to the W-treated control, the percentage of remaining biofilm observed as viable counts after PA treatment was 57 % (SD 16) and after AX treatment 40 % (SD 14). This difference was statistically significant for both PA and AX treatment (*p* < 0.05). After MD treatment viable counts were not significantly lowered, the percentage remaining viable cell counts was 76 % (SD 35) relative to the W-treated control (Fig. 4).

### Organic acids

**Fig. 5:**
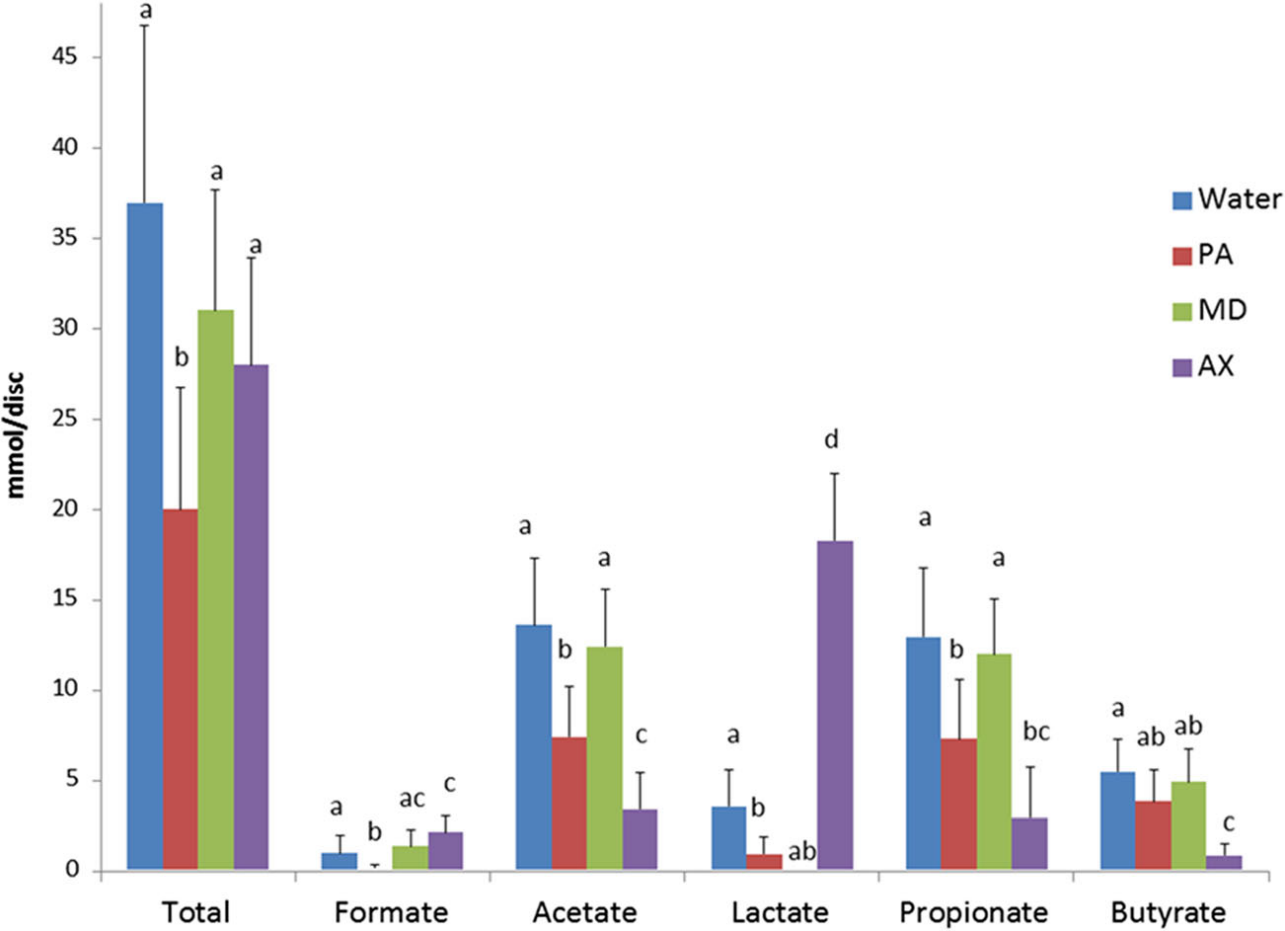
Effect of single treatment on organic acids after a 3-h incubation with 0.2 % sucrose following the respective 10-min treatment of plaque-saliva mixture-derived biofilms. The sum of all acids is represented as total. Different *letters* indicate statistically significant differences between the treatments (*p* < 0.05, one-way-ANOVA, Games-Howel test; *p* value corrected for multiple comparisons) per anion

The concentration of succinate in biofilms was low and similar for all groups (data not shown). PA-treated biofilms had statistically significantly lower amounts of formate, lactate, propionate, and acetate compared to water-treated biofilms after a 3-h incubation with sucrose, while MD-treated biofilms had significantly lower lactate compared to W-treated biofilms (Fig. 5). In contrast, AX-treated biofilms had a significantly higher amount of lactate and significantly lower amounts of acetate, propionate, and butyrate (*p* ≤ 0.001). A positive correlation between the relative abundance of genus *Veillonella* and the sum of acetate and propionate (Pearson *r* = 0.7) excreted to the environment (BPW) was only observed for AX-treated biofilms. When all measured acids were added, PA had a significantly lower amount of total acid than the water control.

## Discussion

In this study, an established microcosm biofilm model was used that allows studying the effects of mouthwashes from a microbiological and ecological perspective. We demonstrated that using this biofilm model, a single exposure to a mouthwash product resulted in different patterns in terms of bacterial composition and metabolic activity of the microcosm biofilm, depending on the chemotherapeutic agent used. Biofilms treated with AX caused major changes in bacterial composition, metabolism, and killing effect. This study did however not evaluate the recovery of the biofilms after a single treatment exposure.

Since the compounds tested in the present study have different mechanisms, we did not attempt to determine the “best antimicrobial” but to characterize their effect with respect to bacterial composition of oral microcosms and their metabolic activity.

While current clinical studies have focused on the amount of plaque and the severity of gingival inflammation, this study highlights the importance of studying not only the “killing” capacity of mouthwashes or chemotherapeutic agents, but also the effect on bacterial composition and metabolic activity The most striking finding was the high amount of lactic acid produced after AX-treatment along with a reduction of the CFUs up to approximately 40 % relative to the water control. This could be explained by the selective inhibition properties of this oxygenating compound [7]. This is further supported by the observation that after AX treatment the relative abundance of genus *Veillonell*a was significantly lower in the treated biofilm accompanied with the higher relative proportion of other genera mainly *Streptococcus*. Increase of streptococci on the other hand, is attributable to the proportionality of the data, because within 10 min, no absolute increase that would imply bacterial growth is possible. Therefore, the only explanation for this finding is that relative abundance of genus *Streptococcus* increases due to a decrease in other taxa such as *Veillonella*.

*Veillonella* which has an important role in biofilm development [26], is a gram-negative bacterium that lacks glucokinase and fructokinase. It is therefore unable to metabolize carbohydrates [26] and instead uses lactate as energy source. The fact that the *Veillonella* proportion was significantly reduced could explain the high lactate amount present in the AX-treated biofilms. These results are in line with the notion that the interaction of these genera can affect the metabolic activity reflected in lactate utilization by biofilms [26], which can potentially affect microbial community development [27].

Although both, PA- and AX-treatment resulted in a lower production of propionate, acetate, and butyrate, this effect was greater for AX-treatment. Butyrate is mainly produced by proteolytic anaerobic bacteria [28, 29] and has been associated with the inflammation found in periodontal disease [30]. AX has the potential to contribute to the treatment of periodontal diseases by further reducing the concentration of acetic, propionic, and butyric acid and by increasing the lactic acid within the GCF. In this way, AX can contribute to the resilience of the subgingival microbiome towards disease. On the other hand, the possible consequences of the higher concentrations of lactate triggered by the AX-treatment need to be elucidated. When produced by probiotics, lactic acid has been described as a natural antimicrobial that is beneficial as an adjunctive to the treatment of periodontitis [31]. Although the results achieved here with AX are promising, in vivo studies are needed to assess this potentially beneficial effect on the non-surgical treatment or in the maintenance of periodontal diseases.

MD-treatment proved to have the ability to inhibit the lactic acid formation by the biofilm without significantly reducing the total viable counts. Our findings that MD significantly lowered the relative abundance of genus *Streptococcus* and the lactate production are in agreement with previous in vitro studies [11].

In this study, we supplemented the growth medium with heated inactivated serum. This was to simulate gingivitis, prevalent in large part of health adult population [2]. Serum is an important component of the gingival crevicular fluid, and the use of 5 % serum in the medium may have influenced the growth of bacteria. Being aware that in the oral environment there is a continuous interaction between supragingival plaque and saliva, we have used this mixture as inoculum. Considering as well, that after a single episode of tooth brushing, on average plaque scores are reduced by only 42 % the plaque that remains on the tooth surfaces [2] will lead to a recolonization of the cleaned areas.

Using the same biofilm model as in the present study, a recent paper describes using saliva from a single donor as inoculum for the production of biofilms, with an initial incubation period of 8 h and a total incubation period of 48 h [19]. It was observed that the composition of the water-treated control biofilms (PMA treated) was dominated by genera *Streptococcus* (62 %), *Veillonella* (35 %), and *Prevotella* (2 %). In the present study, we attempted to increase the diversity of the biofilms by using as inoculum source a mixture of saliva and dental plaque. Furthermore, we increased the incubation period to 24 h and the total incubation period to 96 h, providing time to the slow-growing bacteria to establish within the biofilms. Figure 2 illustrates the relative abundance of the biofilms per donor being the water-treated control biofilms dominated by genera *Veillonella*, *Streptococcus*, *Megasphaera*, *Peptostreptococcus*, and *Prevotella* with 4–20 % of the reads belonging to other genera.

Rudney and collaborators [13] have shown that repeated plaque or saliva inoculums taken on different weeks, from the same donor, clustered together, suggesting that the microbial composition of the biofilms was consistent within subjects over time. The authors have shown that microcosms were relatively stable within subjects over time, but there clearly were differences in species composition between subjects. Subsequently we decided to use multiple donors.

Regarding the variability among donors, it was not only biofilms from the same donor that clustered together, but also the bacterial profiles after the mouthwash treatment. In particular, biofilms derived from donor 11 (Fig. 2) seemed not to be affected by any mouthwash agent as compared to the water treatment. Also where donor 10 had minor changes after PA and MD treatment, it seemed not to be affected by AX as compared to water treatment. Our results confirmed the variation among donors in in vitro biofilms and are in agreement with previous studies [12, 32–34]. Our study supports the notion that pooling samples and averaging the data can mask the individual variability of results of the treatment and the conclusions that are based on them [32, 33]. The differences observed between the biofilms and their inoculum source are in agreement with previous in vitro studies [34, 35].

A recent study has shown differences in bacterial composition at the site level [36]. Whether these differences found in vivo will lead to different microcosms derived from specific niches, and with-in or between donors still needs to be investigated. It is plausible that individual samples from certain sites in a given individual will lead to different results in bacterial composition.

A limitation of our study is that we assessed the effects of a single exposure to mouthwashes, whereas multiple exposures to a chemotherapeutic compound in vitro are known to lead to different results in viability [37]. This could explain in part, why in a previous clinical study we observed different changes in bacterial composition after twice-daily exposures for a week to AX [7]. Oxygenating agents such as AX are on the market while scientific evidence for their use is underreported [4]. Therefore, we underline the need of long-term randomized controlled trials testing these compounds. Concerning “adjunctive” compounds for plaque and gingivitis control such as AX, the American Dental Association (ADA) requires an evaluation period of at least 4 weeks (ADA 1997, 2008) and for mouthwash products, an evaluation period of 6 months.

All studied mouthwashes affected the in vitro biofilms differently. The effects of the AX treatment were the most prominent which resulted in changes of the bacterial composition, metabolism, and in addition affected viability.
